# Uncommon Presentations of Multiple Myeloma

**DOI:** 10.7759/cureus.8400

**Published:** 2020-06-01

**Authors:** Praveena Tathineni, Ivan Cancarevic, Bilal Haider Malik

**Affiliations:** 1 Internal Medicine, University of Illinois at Chicago/Advocate Christ Medical Center, Chicago, USA; 2 Internal Medicine, California Institute of Behavioral Neurosciences and Psychology, Fairfield, USA

**Keywords:** multiple myeloma, malignant hematology, proptosis, pancreatitis, glomerulonephritis, vertigo, plasma cells

## Abstract

Multiple myeloma is a rare malignancy that exhibits a wide range of possible clinical presentations. In recent years, with the advent of stem cell transplantation, the prognosis of patients with multiple myeloma has been increasing. We searched the literature for reports of atypical myeloma presentations to aid clinicians in formulating differential diagnoses and to increase the number of cases diagnosed early. There have been a number of reports of early ocular symptoms, including, but not limited to, proptosis, optic neuropathy, vision loss, retinal hemorrhage, and detachment. Neurological presentations included cranial nerve palsies, vertigo related to cerebellar involvement, and diabetes insipidus related to pituitary involvement. Among gastrointestinal manifestations, there are a number of reports of multiple myeloma presenting as acute and chronic pancreatitis. Mesenteric ischemia due to amyloidosis, acute abdomen, and hepatosplenomegaly were also among reported presentations. When it comes to renal involvement, while acute renal failure and proteinuria are typical, there are reports of patients presenting with both nephritic and nephrotic forms of glomerular disease, as well as end-stage renal disease requiring dialysis. We believe that it is essential for clinicians to keep reporting atypical multiple myeloma presentations and consider it as a possible diagnosis in a patient with serious, atypical symptoms.

## Introduction and background

Multiple myeloma is a malignancy of plasma cells, with an incidence of approximately 30,000 new cases per year in the United States [1,2]. Worldwide, it accounts for approximately 1% of all cancers diagnosed [3]. It is also the most common primary bone cancer among patients older than 70 years [4]. It is rare among young adults, and the median age at diagnosis is 66 years [5]. Due to variable clinical presentations, it is often challenging to diagnose. Hypercalcemia, anemia, renal impairment, and bony pain are among the most common presenting symptoms [6]. Up to three-quarters of patients have anemia at initial presentation, with elevated beta-2-microglobulin levels, localized band on serum protein electrophoresis, X-ray abnormalities, and monoclonal light chains in the urine being frequent [5]. Interestingly, hypercalcemia and creatinine elevations were initially present in less than 20% of patients [5]. The most common early finding was monoclonal protein on immunoelectrophoresis or immunofixation (93%) [5]. Diagnosis generally requires taking a detailed patient history, performing a physical examination, and ordering blood, urine, and radiographic studies followed by performing a bone marrow biopsy [3,7]. Patients most frequently complain of fatigue, pain, decreased physical and cognitive functioning, constipation, and tingling in the hands/feet [8]. Confirming the presence of a monoclonal paraprotein further supports the diagnosis [6]. In order to facilitate early diagnosis of multiple myeloma, the International Myeloma Working Group (IMWG) recommended using clonal plasma cell percentage in the bone marrow, serum-free light chain ratios, and MRI focal lesions as additional biomarkers for the disease [9].

Multiple myeloma is still generally perceived as an incurable disease despite the recent advancements in management. In 2018, it was estimated that more than 12,000 people in the United States died from the disease [2]. Between 1985 and 1998, the median survival remained unchanged at 33 months from the time of diagnosis [5]. Recently, high-dose chemotherapy and autologous stem cell transplant have become the standard of care for patients under the age of 70 years [3,7]. For those who are not candidates for such aggressive treatment, more conventional chemotherapy remains an option [3,7]. In addition, all patients require symptomatic treatment [3,7].

The purpose of this article is to explore some of the less common presentations of multiple myeloma. Taking into consideration the variable presentation of the disease, the significant morbidity and mortality associated with it, and the availability of novel treatments for those diagnosed early, we believe it is important for clinicians to be aware of which patients may need further screening for multiple myeloma.

## Review

Search strategy

The search was conducted using only the PubMed database. The following keywords were initially used: multiple myeloma, plasma cells, and malignant hematology. We have screened the available articles for those that discussed presenting symptoms of multiple myeloma and excluded any studies where the presenting symptoms were those most frequently reported in textbooks, namely proteinuria, bone pain, confusion, fatigue, and infections. It was then followed by a search using some of the rarer initial symptoms that were encountered as keywords, including proptosis, vertigo, glomerulonephritis, and pancreatitis. We decided to use the articles that described the clinical presentations that differed from what is commonly described and where multiple myeloma was not among the initially considered differential diagnoses.

Eye/orbital disease

There is a number of ways in which multiple myeloma can affect different structures within the orbit. The mechanisms of orbital involvement do not generally differ from those of the involvement of the other parts of the body. Pradhan and Custer reported a case of an elderly patient who presented with bilateral tearing secondary to infiltrative lesions of the lacrimal sacs [10]. Malik et al. described a case of bilateral orbital proptosis in a 58-year-old man as an initial presentation of multiple myeloma [11]. CT scan revealed multiple soft tissue lesions, but the skeletal survey did not reveal bony involvement [11]. Tahiliani et al. also reported proptosis as the initial finding [12]. Lam and Rodger reported a case of a 59-year-old woman who presented with bilateral macular detachment, venous stasis retinopathy, and retinal hemorrhages, whereas Brody et al. reported a case of a 64-year-old diabetic male who presented with bilateral exudative macular detachments despite no evidence of diabetic retinopathy [13,14]. Mansour and Salti described a case of multiple myeloma presenting with optic nerve compression [15]. Hogan et al. reported a case of a 39-year-old male who presented with unilateral vision loss caused by a plasmacytoma in the sphenoid sinus leading to optic nerve compression and atrophy [16]. Others have also concluded that proptosis is the most common presenting sign of orbital myeloma [17].

Overall, while clearly not among the most frequent initial manifestations of multiple myeloma, orbital signs have been reported on a number of occasions, with proptosis being the most common. Lacrimal and retinal signs have been reported as well. It seems reasonable that unexplained proptosis, especially in middle-aged and elderly patients, can be considered an indication for multiple myeloma workup. Including multiple myeloma in the workup of non-specific ocular and orbital signs and symptoms is also worth considering, although given the rarity of such presentations, routinely evaluating for myeloma in patients presenting with the retinal or lacrimal disease would likely not be cost-effective.

Gastrointestinal disease

Gastrointestinal signs and symptoms are also not considered classic for multiple myeloma, although there are multiple plausible mechanisms by which myeloma could affect the digestive system, including hypercalcemia and amyloid deposition. Mishra et al. reported a case of a 36-year-old male whose initial manifestation of multiple myeloma was an episode of acute pancreatitis [18]. McIntosh et al. found a similar case in a 22-year-old pregnant woman who also had evidence of preeclampsia, nephrolithiasis, and renal insufficiency [19]. There have also been cases of more chronic pancreatic involvement. Wang et al. reported a case of a 59-year-old man with an eight-month history of fatigue and a two-month history of abdominal pain that was eventually found to have multiple myeloma [20]. Asadi described a case of a 53-year-old woman who presented with a four-month history of abdominal pain, which was eventually caused by small bowel mesenteric amyloidosis in the context of multiple myeloma [21]. Space-occupying liver lesions, plasmacytoma of the spleen, hepatosplenomegaly, dysphagia, and acute abdomen have also been reported as initial myeloma presentations [22-26].

The previously mentioned case reports clearly represent a wide variety of possible manifestations of multiple myeloma. Pancreatic involvement seems to have been most commonly reported, although the tendency of multiple myeloma to cause amyloidosis and progress to plasmacytoma has also led to reports of hepatic, splenic, and mesenteric involvement. A systematic literature review of early gastrointestinal manifestations of multiple myeloma would be helpful in determining how important of a role multiple myeloma should play in the differential diagnosis of otherwise unexplained gastrointestinal problems, especially pancreatitis. We would strongly encourage clinicians to keep reporting any unusual presentations of multiple myeloma.

Neurological disease

While the first symptoms of the central nervous system (CNS) are extremely uncommon for multiple myeloma, compression of brain structures and isolated nerve palsies have been revealed as significant neurological manifestations of the hematological disease. Pika et al. reported a case of a 78-year-old female whose initial manifestation of multiple myeloma was progressively worsening vertigo with nausea, gait instability, and headaches for three months [27]. CT and MRI of the head revealed a large right-sided cerebellar tumor, causing expansion of the cranial vault and compression of surrounding brain structures [27]. Extirpation and histopathological examination of the tumor, along with detailed clinical examination that revealed osteolytic involvement of the skeleton, deemed multiple myeloma as the final diagnosis [27]. Tahiliani et al. described the case of a 40-year-old female who presented with left-sided hemiparesis, later found to have stage III extramedullary multiple myeloma with CNS involvement [12]. Paul et al. reported a case of a 52-year-old male with prolonged low-grade fever, hypernatremia, polyuria, and drowsiness, eventually found to have multiple myeloma infiltration of the posterior pituitary, thus causing cranial diabetes insipidus [28]. Lastly, numerous cases with the diagnosis of multiple myeloma have presented as isolated cranial nerve palsies, specifically oculomotor nerve, trigeminal nerve, and abducens nerve [29-31]. Common initial symptoms of these patients with cranial nerve palsies were diplopia, facial hypoesthesia, and headaches [29-31].

It has been hypothesized that multiple myeloma's attributes, including hyperviscosity, light chain deposits, tendency to cause metabolic abnormalities, and ability to manifest into vasculitis, may explain the disease state's involvement of the CNS [32]. Although neurological symptoms are not the most commonly encountered initial presentation of multiple myeloma, the aforementioned case reports are a clear demonstration of the diverse possibilities of the disease state. It would be prudent for clinicians to keep multiple myeloma on their differential diagnosis, especially in middle-aged and elderly patients presenting with isolated cranial nerve palsies.

Renal disease

Renal signs and symptoms are considered rather classic for multiple myeloma; however, the prototypical hypercalcemia and acute kidney injury are not the only harbingers of disease. Maturana-Ramírez et al. described the case of a 73-year-old female who initially presented with weight loss and macroglossia, initially diagnosed with amyloidosis of the tongue [33]. Further medical evaluation revealed hypercalcemia, hyperphosphatemia, and end-stage renal disease requiring emergent hemodialysis, with the final diagnosis being systemic amyloidosis with multiple myeloma [33]. Oweis et al. reported a case of a 48-year-old man whose initial manifestation of multiple myeloma was focal segmental glomerulosclerosis [34]. Sadeghi and Akbari described the case of a 44-year-old male diagnosed with multiple myeloma, who initially presented with lower extremity edema and was thought to have idiopathic membranous glomerulonephritis, as well as focal tubulointerstitial nephritis [35]. Various types of glomerulonephritis have been associated with the first presentation of multiple myeloma, including fibrillary glomerulonephritis, mesangial proliferative glomerulonephritis, and even crescentic rapidly progressive glomerulonephritis [36-40].

Renal involvement in multiple myeloma is multifactorial. The most common cause of proteinuria in myeloma is light chain proteinuria, often leading to a concurrent diagnosis of amyloidosis [35]. Myeloma kidney is a renal emergency, and physicians must treat patients accordingly and immediately. As demonstrated by the case reports mentioned above, glomerulonephritis is also an initial manifestation of multiple myeloma. It is important to keep in mind that both nephritic and nephrotic pathologies can lead to a diagnosis of multiple myeloma. The extent of acute kidney injury varies widely in myeloma patients, and it is highly recommended that clinicians work up renal abnormalities no matter what the severity of renal damage, especially in patients with concurrent hypercalcemia and/or proteinuria.

## Conclusions

Multiple myeloma is a relatively rare plasma cell malignancy. Although hypercalcemia, bone pain, and renal impairment are the classically reported early findings, the actual clinical presentation of this disease is highly variable. The involvement of the orbits and the eye was reported on a number of occasions, with proptosis being the most frequent ocular manifestation of multiple myeloma. Retinal detachment, hemorrhage, optic nerve compression, and unilateral vision loss due to plasmacytoma were also reported. Multiple myeloma can also present as a gastrointestinal disease. Pancreatic involvement, both acute and chronic, was reported in a number of articles. Mesenteric ischemia, acute abdomen, hepatosplenomegaly, space-occupying lesions, and dysphagia were among other reported early manifestations. The neurological presentations have included pituitary involvement leading to diabetes insipidus, cerebellar involvement causing vertigo, and cranial nerve involvement leading to palsies. There have also been many reports of highly atypical renal presentations, including different forms of glomerulonephritis, such as focal segmental glomerulonephritis and membranous glomerulonephritis, and even end-stage renal disease. Both nephritic and nephrotic presentations have been reported. Considering the morbidity and mortality associated with multiple myeloma and the wide range of possible presentations, we believe it is important for clinicians to keep reporting atypical presentations.
